# Development of Reverse Transcriptase Recombinase Polymerase Amplification Combined with Lateral Flow Dipstick for Rapid Detection of Tilapia Lake Virus (TiLV): Pilot Study

**DOI:** 10.3390/vetsci12090845

**Published:** 2025-09-01

**Authors:** Saralee Srivorakul, Thunyamas Guntawang, Tidaratt Sittisak, Thanchanok Gordsueb, Kittikorn Boonsri, Rutch Khattiya, Nattawooti Sthitmatee, Kidsadagon Pringproa

**Affiliations:** 1 Division of Veterinary Sciences, Faculty of Veterinary Medicine, Chiang Mai University, Chiang Mai 50100, Thailand; saralee.s@cmu.ac.th (S.S.); thunyamas.g@cmu.ac.th (T.G.); tidaratt_s@cmu.ac.th (T.S.); thanchanok_go@cmu.ac.th (T.G.); rutch.k@cmu.ac.th (R.K.); nattawooti.s@cmu.ac.th (N.S.); 2 Center of Veterinary Medical Diagnostic and Animal Health Innovation, Faculty of Veterinary Medicine, Chiang Mai University, Chiang Mai 50100, Thailand; kittikorn.boonsri@cmu.ac.th

**Keywords:** TiLV, recombinase polymerase amplification, lateral flow dipstick

## Abstract

Tilapia Lake Virus (TiLV) is well known as a highly contagious viral infection in aquaculture, particularly affecting Tilapia worldwide. Until recently, various TiLV diagnostic methods have been used for rapid and accurate diagnostic procedures that are crucial for timely disease detection and reducing losses. In this study, we developed an alternative method for investigating TiLV diagnosis using Reverse Transcriptase Recombinase Polymerase Amplification (RT-RPA) assay combined with a lateral flow dipstick (LFD). The test was generated by specific anti-FITC and anti-Biotin capture antibodies that are compatible with the TiLV-specific primers tagged with FITC and Biotin. The test was conducted by the reverse transcriptase of target TiLV RNA and RPA amplification at 39 °C for 20 min. The products were then determined by a positive band signal via LFD. The RT-RPA-LFD assay detected the plasmid of TiLV (pTiLV) with a Limit of Detection (LOD) of 3.19 copies/µL, while the RT-PCR-LFD assay detected it with an LOD of 319 copies/µL. Our findings demonstrate that RT-RPA-LFD represents a possible alternative to RT-PCR for the rapid and sensitive detection of TiLV, especially in areas with limited infrastructure.

## Simple Summary

Tilapia Lake Virus (TiLV) is a significant viral pathogen affecting tilapia, a key species in global aquaculture. In this study, we developed a rapid and user-friendly diagnostic assay for TiLV detection, utilizing reverse transcription recombinase polymerase amplification (RT-RPA) in combination with a lateral flow dipstick (LFD). This approach enables amplification of the viral genome at a constant temperature and provides a visual result on a paper strip within approximately 20 min. The assay demonstrates a detection sensitivity approximately 100 times greater than that of a comparable RT-PCR method. Notably, the technique does not require complex laboratory instrumentation, making it highly suitable for field applications. This test offers a practical tool for fish farmers and aquatic animal health professionals to identify TiLV infections promptly and implement timely management interventions.

## 1. Introduction

Tilapia Lake Virus (TiLV) is known as a highly contagious virus affecting Tilapia, a key species in aquaculture. TiLV outbreaks were first reported in Israel in 2014 and revealed a high mortality rate among tilapia across wild and farm aquaculture [1]. Since then, TiLV has been recognized as one of the most important pathogens that has caused economic losses in aquaculture worldwide. Presently, TiLV has been reported in various countries including Israel, Egypt, Ecuador, Uganda, Tanzania, Peru, Mexico, the United States, India, Bangladesh, Malaysia, Indonesia, Taipei, the Philippines, and Thailand [2]. It is well accepted that fingerlings, which are recognized as highly susceptible hosts, are often exposed to TiLV when being transferred to new ponds and have exhibited high mortality rates of up to 90% [3,4]. The species that are most likely to be infected by TiLV are Nile tilapia (*Orechromis niloticus*), red tilapia (*Oreochromis* spp.), blue tilapia (*Oreochromis aureus*), Mozambique tilapia (*Oreochromis mossambicus*), hybrid tilapia (*O. niloticus* × *O. aureus* or *O. niloticus* × *O. mossambicus*), *Tilapia zilli*, *Sarotherodon galilaeus*, and *Tristamellasimonis intermedia* [2,5]. Viral TiLV particles can be introduced into the fish through direct contact with gills or via the oral route. The virus then disseminates to the liver, spleen, anterior kidneys, intestines, or gonads [2,6,7]. Ultimately, the virus can be shed via mucus secretion and infect eggs [2]. Clinically, infected tilapia tend to display the clinical signs of skin redness, erosion, ulcers or hemorrhages of the skin, exophthalmia, brain congestion, hepatomegaly, and pale liver disease [1,2,8,9,10]. Microscopic lesions can include necrotic splenitis, necrotic nephritis, syncytial giant cells in the liver, or lymphocytic perivascular cuffing in the brain [1,9,10,11,12,13].

Typically, TiLV can be effectively diagnosed through various techniques. Conventional Reverse Transcriptase Polymerase Chain Reaction (RT-PCR) and Reverse Transcriptase quantitative PCR (RT-qPCR) are extensively employed owing to their high specificity and sensitivity. However, their implementation necessitates well-equipped laboratory infrastructure and proficient personnel, thereby restricting their use in resource-limited settings. This limitation also applies to related techniques such as Nested RT-PCR, Semi-nested RT-PCR, and Digital PCR (dPCR). Notably, RT-qPCR offers notable advantages through its enhanced sensitivity and capacity for viral load quantification. Simple alternative methods that operate under constant temperatures and have been studied in relation to TiLV detection are Reverse Transcriptase Loop-Mediated Isothermal Amplification (RT-LAMP), Reverse Transcriptase Recombinase Polymerase Amplification (RT-RPA), and RT-RPA-Cas12a. These methods were developed most recently and have been explored in terms of rapid, sensitive, and equipment-independent assay that can be reliably used for on-site detection of TiLV. Additional methods are cell culture, necropsy, histopathology, in situ hybridization (ISH), immunohistochemistry (IHC), and indirect ELISA [5]. The present study addresses this gap by developing and evaluating an RT-RPA-LFD assay that combines speed, sensitivity, and minimal equipment requirements.

**Figure 1:**
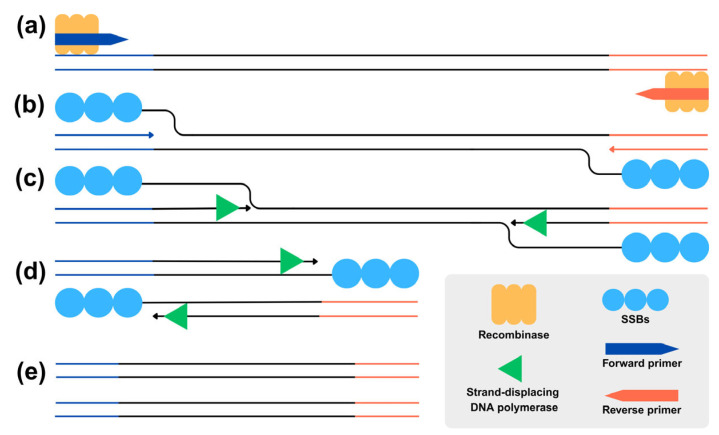
Schematic diagram of RPA reaction. (**a**) Recombinase enzymes bind to oligonucleotide primers and form recombinase–primer complexes. (**b**) SSBs bind to displaced DNA strands and form D-loops. (**c**) Strand-displacing DNA polymerase initiates synthesis and extends DNA strands. (**d**,**e**) Cycle continues under isothermal amplification, resulting in exponential accumulation of target nucleic acid sequence.

RPA is a molecular biology technique that is principally based on isothermal amplification of the target gene using certain enzymes. The enzymatic components involved in the reaction include Recombinase, single-stranded DNA binding proteins (SSBs), and strand-displacing DNA polymerase [14]. The reaction was initiated by binding the Recombinase enzymes to oligonucleotide primers including forward and reverse primers (Figure 1a), together with other components such as recombinase-loading factors, salt molecules, crowding agents, and energy and fuel components [14]. These complexes possess the ability to scan double-stranded DNA for sequences complementary to the primers and facilitate the invasion of the primer into the homologous region of the target double-stranded DNA. The SSBs subsequently bind to the displaced DNA strand, forming a displacement loop (D-loop) that prevents reannealing and stabilizes the single-stranded region (Figure 1b). Consequently, a strand-displacing DNA polymerase initiates synthesis and extends the DNA strand under isothermal amplification (Figure 1c). This cycle is continuously repeated, leading to the exponential amplification of the target nucleic acid sequence (Figure 1d,e). It is well established that the RPA reaction requires a constant low temperature, typically maintained between 37 °C and 42 °C. The process is often completed within 20 to 40 min [14,15,16]. The RPA product can then be visualized after undergoing 1.5–2% agarose gel electrophoresis or lateral flow dipstick (LFD) assay. During the RPA-LFD process, the primers used in the reaction are usually tagged with Biotin, while probes are labelled with fluoresceine such as FAM or FITC [14,17]. The amplified products coupled with the primers are then visually detected on a lateral flow strip. This easy, short, and highly sensitive method is ideal for the diagnosis of various pathogens such as Feline Leukemia virus [16], African Swine Fever virus [18], Cyprinid Herpesvirus 2 virus [15], epizootic hemorrhagic disease virus [19], or *Elizabethkingia miricola* [20]. As RPA assay has been applied in TiLV detection as RT-RPA-Cas12a that targeted TiLV segment 9 [21] and real-time RPA assay that targeted TiLV segments 1 and 2 [22], it represents an effective diagnostic alternative to RT-PCR. While conventional RT-PCR necessitates time-consuming gel electrophoresis for result visualization, limiting its field applicability, RT-RPA imposes distinct requirements such as greater primer specificity and extensive optimization, and often necessitates product purification prior to electrophoretic analysis. Therefore, in the present study we have demonstrated the use of an RPA-based assay combined with a lateral flow dipstick (LFD) to enhance the accessibility and feasibility of TiLV diagnosis in field situations.

## 2. Materials and Methods

### 2.1. Tissue Preparation

Tissue samples obtained from Tilapia that had previously been identified to be positive or negative for TiLV were RNA extracted using a Nucleospin^®^ RNA kit (Macherey-Nagel, Dueren, Germany). They were then synthesized for cDNA using a ReverTra Ace™ qPCR RT Master Mix (Toyobo, Osaka, Japan), according to the manufacturer’s instructions. Lastly, the cDNA samples were kept at −20 °C until use.

### 2.2. Primer Design

The primers that targeted segment 3 of TiLV were designed, synthesized, custom tagged with Fluorescein Isothiocyanate (FITC) in the forward primer (TiLV-112F: 5′-FITCCTGAGCTAAAGAGGCAATATGGATT-3′), and tagged with Biotin in the reverse primer (TiLV-112R: 5′-BiotinCGTGCGTACTCGTTCAGTATAAGTTCT-3′). The full set of primers was analyzed for qPCR according to previous studies, with an expected product size of 112 bp [23,24]. The length of the primers should be between 28 and 30 nucleotides, which is consistent with what has been recommended for the RPA protocol.

### 2.3. RT-PCR for Primer Screening

The TiLV primer was processed to optimize the RT-RPA-LFD assay and was comparable with RT-PCR. The RT-PCR mixture was established by using Quick Taq™ HS DyeMix (Toyobo, Osaka, Japan) at a total volume of 20 µL. Briefly, the template DNA was immersed in 1 µL mixed with 10 µL of 2X QuickTaq™ HS DyeMix (Toyobo, Osaka, Japan), along with 0.2 µL of the forward and reverse primers, while DEPC water was added to determine the final volume. The PCR cycling conditions consisted of a pre-denaturation step at 95 °C for 5 min, followed by 35 cycles of denaturation at 95 °C for 30 s, an annealing step at 55 °C for 30 s, and an extension step at 72 °C for 30 s. The final extension step was then administered at 72 °C for 10 min. Ultimately, gel electrophoresis via 2% agarose gel was established for visual result detection.

### 2.4. Development of RT-RPA-LFD Assay

The custom primer set described above was used to process RPA amplification using the TwistAmp^®^ Basic kit (TwistDx, Cambridge, UK). Briefly, the mixture used for the RPA reaction was composed of 1 µL of template DNA, 2.4 µL of the forward and reverse primers, 29.5 µL of the rehydration buffer, and 12.2 µL of dH_2_O. A total volume of 47.5 µL was then combined with 2.5 µL of 280 mM magnesium acetate, followed by immediate incubation in a heat block at 39 °C for 20 min. RPA purification was achieved with a QIAquick^®^ PCR and Gel Cleanup Kit (Qiagen, Dusseldorf, Germany) before the LFD test or gel electrophoresis was carried out.

**Figure 2:**
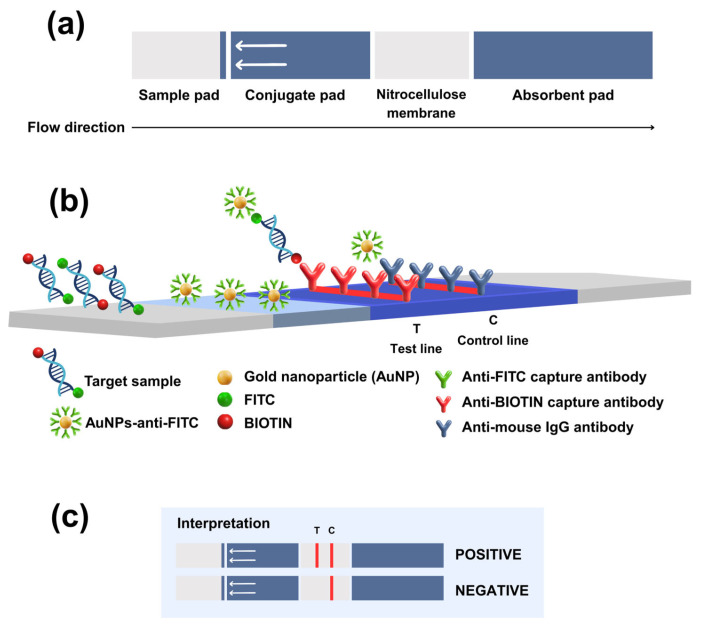
Schematic diagram of lateral flow dipstick (LFD) assay; (**a**) LFD was composed of four parts including sample pad, conjugate pad, nitrocellulose membrane, and absorbent pad. (**b**) Amplified samples were mixed with DNA running buffer and applied to sample pad. AuNPs-anti-FITC–DNA complex was run through strip and captured with anti-Biotin capture antibody in test line, while AuNPs-anti-FITC was captured with anti-mouse IgG antibody in control line. (**c**) Amplified samples were applied onto the sample pad at the location indicated by the arrow. TiLV-positive samples were represented by two lines, while TiLV-negative samples showed only control line.

The LFD was generated and produced by K biosciences Co., Ltd., Pathumthani, Thailand. Each LFD test was composed of four overlapping parts including a sample pad, a conjugate pad, a nitrocellulose membrane, and an absorbent pad that allowed fluid to migrate by capillary diffusion (Figure 2a). In this study, the TiLV RPA-LFD assay was constructed (Figure 2b), wherein the target sequence was first amplified by RPA using primers labeled with Fluorescein Isothiocyanate (FITC) and Biotin. Briefly, the target sample was mixed with running buffer (1:100), and then a sample pad of LFD was dipped into the mixed solution for at least 30 s. After that, the fluid was allowed to pass via capillary diffusion through the conjugate pad for 15 min. In this conjugate pad, where the gold nanoparticles (AuNPs) were coated, the AuNPs-anti-FITC probe was applied to bind with the FITC tag on each amplicon via antibody–antigen interactions, resulting in AuNPs-anti-FITC–DNA complexes that continued to migrate along the strip. When the complex reached the nitrocellulose membrane, the test line, coated with the immobilized anti-Biotin capture antibody, was captured with the Biotin tag on the other end of the amplicon. These captures produced a visible, red-colored band on the test line. For the control line, excess AuNP–anti-FITC continued to flow onto the control line, which had been coated with anti-mouse IgG antibody. Consequently, a second colored line was observed. Interpretation of the positive signal was determined by the presence of bands on the test and control lines, while a negative signal was indicated by a single band on the control line (Figure 2c).

### 2.5. Determination of the Limit of Detection (LOD)

The LOD of RT-RPA-LFD and RT-PCR-LFD was determined by using a 10-fold serial dilution of plasmid TiLV (pTiLV), which was represented by 3.19 × 10^0^ to 3.19 × 10^6^ copies/µL. A synthetic plasmid was constructed by inserting a 552 bp fragment of the TiLV segment 3 genome (GenBank accession no. KJ605629) into the pMA-T vector and transformed in *E. coli* K12 OmniMAX™ 2T1R (Invitrogen, San Diego, CA, USA). The viral copy number was then calculated by using the following equation:

Copy number = (amount (ng) × 6.022 × 10^23^)/(length of plasmid (bp) × 660 × 10^9^).

### 2.6. Validation LFD in TiLV-Infected Tissues

The TiLV-positive and TiLV-negative tilapia tissue samples that were utilized in this study were obtained from the Center of Veterinary Medical Diagnostic and Animal Health Innovation, Chiang Mai University (CMU), Chiang Mai, Thailand. The cDNA synthesized from these samples served as a template for both RT-RPA and RT-PCR amplification. The resulting DNA products were subsequently analyzed using LFD assays.

## 3. Results

### 3.1. Optimization of the Reaction

**Figure 3:**
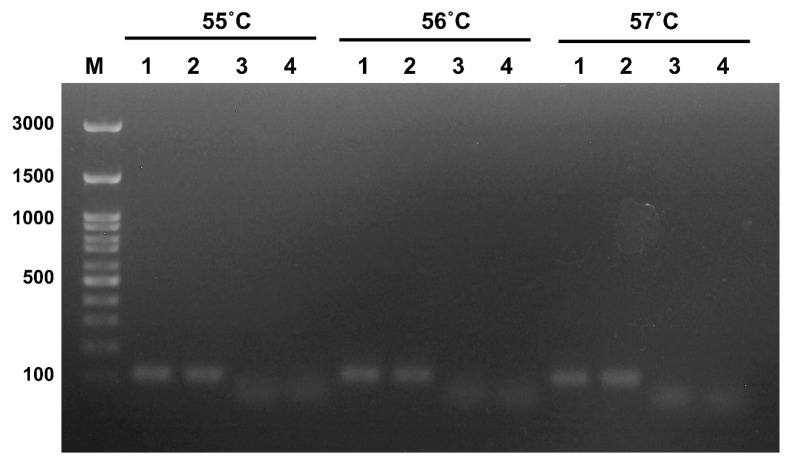
Optimization and validation of primers using RT-PCR. Primers for segment 3 of TiLV were tagged with FITC and Biotin. Optimization of annealing temp was performed using different temperatures of 55 °C, 56 °C, and 57 °C. Results were indicated by clear signals of expected PCR products, as follows: 112 bp at 57 °C. M = DNA marker, 1 = TiLV supernatant, 2 = pTiLV (3.19 × 10^6^ copies/µL), 3 = TiLV negative tissue, and 4 = negative control.

To adjust conditions for the RPA reaction, the primer set was fixed with different annealing temperatures ranging from 55 to 57 °C and tested by RT-PCR. The expected PCR product size was shown to be 112 bp, as has been indicated in lane nos. 1–2, which were representative of TiLV supernatant and pTiLV, respectively (Figure 3). The primers were then further applied for the RPA assay under an amplification temperature of 39 °C for 20 min.

### 3.2. Limit of Detection (LOD)

**Figure 4:**
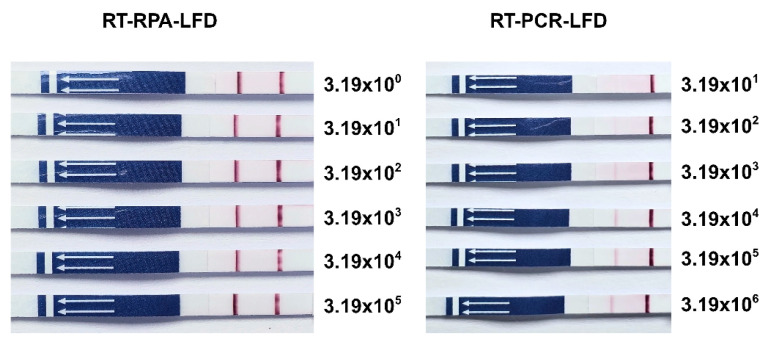
Lateral flow dipstick test for determination of LOD in pTiLV. RT-RPA-LFD detected pTiLV at lowest concentration level of 3.19 × 10^0^ copies/µL, while RT-PCR-LFD detected pTiLV at concentration levels of 3.19 × 10^2^ copies/µL onward.

Plasmids of TiLV (pTiLV) were serially 10-fold diluted and their LOD was determined by either RT-RPA-LFD or RT-PCR-LFD. The results indicated that RT-RPA-LFD had 100 times higher sensitivity than that of RT-PCR-LFD (Figure 4). RT-RPA-LFD demonstrated an LOD composed of 3.19 × 10^0^ copies/µL, while RT-PCR-LFD displayed an LOD composed of 3.19 × 10^2^ copies/µL.

### 3.3. Application of RT-RPA-LFD in Tilapia Tissues

**Figure 5:**
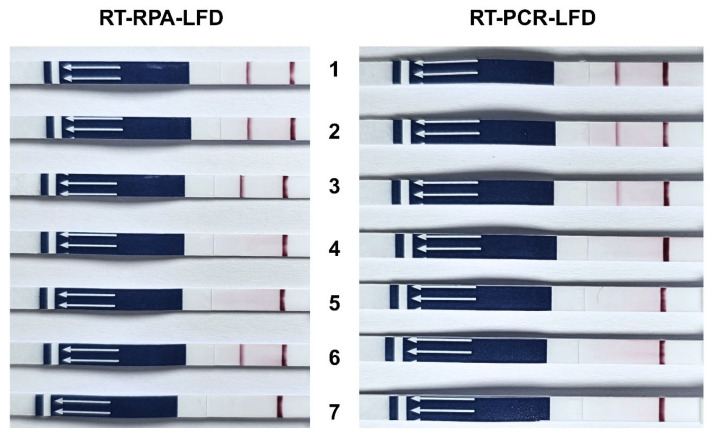
Lateral flow dipstick test of tilapia tissues obtained from amplified products of RT-RPA and RT-PCR. RT-RPA-LFD DNA dipstick demonstrated positive results for sample nos. 1–3 and 6, while RT-PCR-LFD DNA dipstick did so for sample nos. 1–3. Accordingly, sample nos. 1–3 = TiLV true positive tissues, sample nos. 4–7 = TiLV true negative tissues.

To determine whether RT-RPA-LFD can be applied to Tilapia tissues, cDNA from TiLV-PCR-confirmed true positive samples (sample nos. 1–3) and true negative samples (sample nos. 4–7) was subjected to RT-RPA-LFD analysis. As has been shown in Figure 5, products from sample nos. 1–3 generated positive signals on the LFD in both RT-RPA and RT-PCR assays, consistent with their true positive status. Notably, although sample no. 6 tested negative by RT-PCR, the RT-RPA assay yielded a positive LFD signal. This result represents a false positive outcome, thereby reducing the specificity of the RT-RPA assay.

### 3.4. Determination of Specificity of RT-RPA-LFD

**Figure 6:**
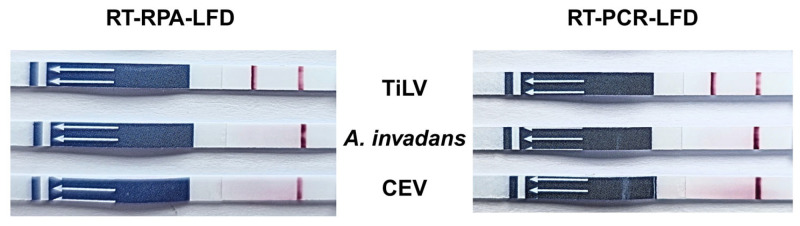
Comparison between RT-RPA-LFD and RT-PCR-LFD lateral flow dipstick tests of fish pathogens. Both RT-RPA-LFD and RT-PCR-LFD assays show positive results for TiLV, while they are shown to be negative for *A. invadans* and CEV.

To evaluate potential cross-reactivity, the RT-RPA-LFD and RT-PCR-LFD assays were tested against other fish pathogens, including *Aphanomyces invadans* and Carp Edema Virus (CEV). Among these, only TiLV yielded a positive result in the RT-RPA-LFD and RT-PCR-LFD assays, whereas the other pathogens were shown to be negative (Figure 6).

## 4. Discussion

The present study demonstrates the development of an RT-RPA-LFD assay for the rapid and sensitive detection of TiLV, with its performance metrics being compared to RT-PCR-LFD. A key finding of this study was the assay’s significant detecting capability at up to 3.19 × 10^0^ copies/µL, which was determined to be more sensitive than RT-PCR-LFD (3.19 × 10^2^ copies/µL). This result revealed the ability of RPA to amplify low viral loads. These outcomes were in accordance with those of previous reports involving the Zika and Ebola viruses, where RPA achieved detection limits as low as 10^−5^ copies/µL [25]. Both RT-RPA-LFD and RT-PCR-LFD exhibited 100% clinical sensitivity, which was consistent with previous reports on RPA-based assays used for other pathogens such as SARS-CoV-2 [26] and Avian Influenza virus [27]. However, RT-RPA-LFD showed reduced specificity (75%) due to a single false positive result, whereas RT-PCR-LFD maintained 100% specificity. Compared with previously reported RPA-based approaches, such as RPA-Cas12a targeting segment 9 [21] and real-time RPA assays targeting segments 1 and 2 of TiLV [22], these methods demonstrated high analytical sensitivity and exhibited no cross-reactivity with other fish pathogens. In contrast, the false positive rate of RT-RPA-LFD observed in this study may be explained by the existence of non-specific primer interactions or the unintended amplification of non-target sequences [17,28]. The set of primers used in this study was designed to amplify a conserved region of the TiLV genome, with certain modifications (5′ FITC and Biotin tags) being employed to enable lateral flow dipstick (LFD) detection. This labeling strategy is widely used in LFD assays and facilitates the visual detection of amplification products on lateral flow strips [29,30,31]. While the current primers enable sensitive RT-RPA TiLV detection due to the rapidly initiated amplification without the need for high-temperature denaturation of recombinase and strand-displacing polymerase enzymes at about 100 copies/μL compared to RT-PCR, their moderate GC content recorded at about 39.3% GC and 36.7% GC could potentially produce non-specific amplification or dimer formation [32].

In this study, a TwistAmp^®^ Basic Kit was employed to develop an RT-RPA-LFD assay for TiLV detection. To adapt the Basic Kit for LFD, primers were modified with a 5′-FITC label and a 5′-Biotin tag, enabling amplicon capture and visualization on the dipstick via anti-FITC and anti-Biotin antibodies. Notably, probe-dependent RPA kits (e.g., TwistAmp^®^ nfo) should be more highly specified to amplify the target samples due to probe cleavage. This process would then cleave a specific probe containing a tetrahydrofuran (THF) and release a labeled fragment for detection [22,29,33,34,35]. However, the TwistAmp^®^ Basic Kit relies on primer labeling (FITC/Biotin), which is more cost effective but also more prone to primer–dimer artifacts than TwistAmp^®^ nfo. This outcome has been seen in the false positives of sample no. 6. Conventional PCR also offers better control over reaction kinetics when compared with isothermal methods such as RPA [36]. These findings are consistent with those of previous reports that emphasize the need for careful primer design and screening when using RPA to reduce nonspecific binding, as well as the adjustment of reaction temperature and time to optimize specificity. This is especially true when employed in combination with lateral flow-based detection to minimize false positive results [17]. In addition, cross-reactivity testing was limited to *Aphanomyces invadans* and Carp Edema Virus (CEV), as these were the only non-TiLV aquatic pathogens available in our laboratory. While these pathogens are not the most common in tilapia, they were used to demonstrate the feasibility of the RT-RPA-LFD assay in detecting TiLV without cross-reactivity. This work was conducted as a pilot study and should extend the evaluation to include a broader range of tilapia-specific pathogens in future research. Regarding cost considerations for practical application, the estimated expense for performing the RT-RPA-LFD assay is approximately 18 to 24.24 USD per reaction, which is comparable to the estimated cost of RT-PCR-LFD at around 18 to 21.21 USD per reaction. It is important to note that the cost estimate for RT-PCR-LFD excludes the additional investment required for thermocycler equipment. In summary, we emphasize that this study serves as a preliminary investigation, and the assay is not yet intended for commercial deployment. Consequently, these cost estimates are provisional and may be revised following further optimization and large-scale validation. Moreover, the limited sample size in this study constrains the generalizability of the findings, particularly in relation to total tilapia tissue samples, other fish pathogens, and environmental specimens commonly associated with tilapia. The misclassification of even a single sample can substantially influence diagnostic performance.

## 5. Conclusions

In conclusion, RT-RPA-LFD offers a rapid alternative to RT-PCR for TiLV detection but requires refinement to mitigate false positives. Continued improvements in primer design, implementation of stringent contamination control measures, and validation with larger sample cohorts to confirm the robustness of sensitivity and specificity are critical for addressing these limitations.

## Data Availability

Data is contained within the article.

## Abbreviations

The following abbreviations are used in this manuscript:

AuNPs	Gold nanoparticles
*A. invadans*	*Aphanomyces invadans*
CEV	Carp Edema Virus
FITC	Fluorescein Isothiocyanate
LFD	Lateral flow dipstick
LOD	Limit of Detection
pTiLV	Plasmid Tilapia Lake Virus
RT-RPA	Reverse Transcriptase Recombinase Polymerase Amplification
RT-PCR	Reverse Transcriptase Polymerase Chain Reaction
TiLV	Tilapia Lake Virus
